# Secretion of polyhydroxybutyrate in *Escherichia coli* using a synthetic biological engineering approach

**DOI:** 10.1186/1754-1611-7-24

**Published:** 2013-10-18

**Authors:** Asif Rahman, Elisabeth Linton, Alex D Hatch, Ronald C Sims, Charles D Miller

**Affiliations:** 1 Department of Biological Engineering, Utah State University, 4105 Old Main Hill, Logan 84322-4105, UT, USA

**Keywords:** Polyhydroxyalkanoates, PHA, PHB, Secretion, Phasin, Translocation, HlyA, Synthetic biology

## Abstract

**Background:**

Polyhydroxyalkanoates (PHAs) are a group of biodegradable plastics that are produced by a wide variety of microorganisms, mainly as a storage intermediate for energy and carbon. Polyhydroxybutyrate (PHB) is a short-chain-length PHA with interesting chemical and physical properties. Large scale production of PHB is not wide-spread mainly due to the downstream processing of bacterial cultures to extract the PHB. Secretion of PHB from *Escherichia coli* could reduce downstream processing costs. PHB are non-proteinaceous polymers, hence cannot be directly targeted for secretion. Phasin, PhaP1, is a low molecular weight protein that binds to PHB, reducing PHB granule size. In this study PHB is indirectly secreted with PhaP1 from *E. coli* via type I secretion using HlyA signal peptides.

**Results:**

This study demonstrated the successful secretion of phasin and phasin bound PHB outside of the cell and into the culture medium. The secretion of PHB was initiated between 24 and 48 h after induction. After 48 h of culturing, 36% of the total PHB produced in the secreting strain was collected in the secreted fraction and 64% remained in the internal fraction. To further support the findings of this study, the PHB secretion phenomenon was observed using SEM.

**Conclusions:**

From this study, the ability to use type I secretion to: 1) secrete phasin and 2) successfully secrete PHB has been shown.

## Background

Fossil derived plastics are non-biodegradable and toxic to the environment. Based on an United States Environmental Protection Agency study in 2011, there was an increase in non-biodegradable plastic accumulation in municipal solid waste systems from 0.5% to 12.4% during 1960 to 2010 [1]. Alternative means of producing plastics in large quantities that are both economically and environmentally friendly have recently gained considerable attention [2].

Replacing traditional plastic with biodegradable plastic such as polyhydroxyalkanoates (PHAs) can potentially reduce total waste by up to 20% [3]. PHAs are produced by a variety of microorganisms as an intercellular storage medium for energy and carbon and can accumulate up to 90% of the cell dry weight [4]. PHAs are biodegradable polymers. The biodegradability can range from days [5] to months [6] with degradation either taking place extracellularly or intracellularly. Extensive review on degradation of PHAs can be found in [7,8]. There are 155 different confirmed types of PHA monomer subunits, each with varying monomer repeat number and side groups [9]. Additionally, PHAs have melting temperatures between 50-180°C and crystallinity of 30–70% [10]. Thus, PHAs have a variety of possible applications, that could replace traditional plastics derived from petroleum [11]. Some of the possible applications are highlighted in previous studies [12,13] and include: packaging, medical uses [14], agricultural uses, and in carbon nanotubes [15].

Polyhydroxybutyrates (PHB) are a short-chain-length (scl) PHA polyester with between 3–5 carbon monomers [9]. The production of PHB in recombinant systems such as *Escherichia coli* has been made possible by the isolation of the *phaCAB* operon from *Ralstonia eutropha* (*Cupriavidus necator*) and cloning into pBluescriptSK- to generate plasmid pBHR68. pBHR68 has been widely used for recombinant production of PHB in *E. coli*. The *phaCAB* operon is a three-step enzymatic process by which acetyl-CoA is converted to PHB: *phaC* (PHA synthase), *phaA* (β-ketothiolase), and *phaB* (acetoacetyl-CoA reductase) [4,16]. After production of PHB, the PHB polymer forms spherical granules with a hydrophobic core and attached proteins at the surface, including PHA synthase and phasin, PhaP1 [17-19].

The cost of producing PHAs is approximately US$ 4-6/ kg and one of the major bottlenecks in scaling up recombinant PHA production systems is the isolation of the PHAs [12,20]. Current techniques that are used to isolate PHB from *E. coli* are invasive, including mechanical, chemical, and biological treatments. These techniques involve lysing the cellular membrane prior to isolation of the PHBs. There are a variety of methods that have been suggested for recovery of PHBs from microorganisms and a detailed review of the different PHB isolation methods is outlined in [20].

The development of a secretion mechanism eliminates the need for cell disruption by mechanical or chemical means, and may lead to continuous (or semi-continuous) PHB production systems. In gram-negative bacteria like *E. coli*, compounds are exported via six major secretory pathways [21-24]. Recombinant proteins can be targeted to type I and II secretory pathways through genetic fusion with signal peptide targeting sequences.

PHBs are non-proteinaceous polyesters and therefore cannot be directly targeted for translocation by signal peptide fusion. Phasins are low-molecular weight proteins that play a role in PHB granule formation by binding to the PHB granule surface [19,25]. Phasins are structural proteins found in organisms that naturally produce PHAs and are similar in function to oleosins which are found in plants [19,26]. Currently, oleosins are used to purify various compounds such as pharmaceuticals from plants [27].

Translocation of PHBs is possible through optimization of granule size, which reportedly varies from 50 to 1,000 kDa based on growth parameters and host strain [4]. One of the functions of phasin is to increase the surface-to-volume ratio of granules so that higher accumulation levels can be achieved [25,28,29]. Therefore, the size of the PHB granule can be decreased significantly through phasin overexpression. In one such study, overexpression of phasin resulted in a decrease of PHB granules from 70–310 nm diameter to 20–60 nm [28].

Type I secretion is a simple one step secretion system that can translocate proteins from the cytoplasm to the extracellular medium without protein interaction with the periplasm [30]. Proteins of nearly 900 kDa (large adhesion protein, *LapA*) have reportedly been secreted to the extracellular milieu by the type I secretory mechanism of gram-negative bacteria [31,32]. Specifically using the Hemolysin (HlyA) secretion mechanism proteins such as β-galactosidase (117 kDa) [33], β-gal-OmpF (56 kDa) [34], and green fluorescent protein (GFP) (27 kDa) [35] have been secreted by *E. coli*. The physical characteristics of the secretion channel are approximately 3.5 nm in diameter with a length of 14 nm as reported by Fernández et al., which makes the secretion phenomena of large proteins very interesting [36].

Our group has previously used a synthetic biological engineering approach to demonstrate the feasibility of HlyA, GeneIII, PelB, and TorA secretion systems in *E. coli* with the use of GFP [35]. From the aforementioned study, the type I secretion system using the HlyA signal peptide yielded the best results for secretion of GFP outside of the cell and into the medium [35]. The objective of this study was to demonstrate that phasin can be used to secrete PHB from *E. coli* using type I secretion machinery.

## Results and discussion

Initial studies were carried out to demonstrate expression and then successful translocation of phasin, PhaP1, into the extracellular medium. Once this was demonstrated, PHB secretion experiments were conducted that included: growth studies, PHB production in secreted and non-secreted fractions, and visualization with scanning electron microscopy (SEM).

### Analysis of phasin translocation

**Figure 1:**
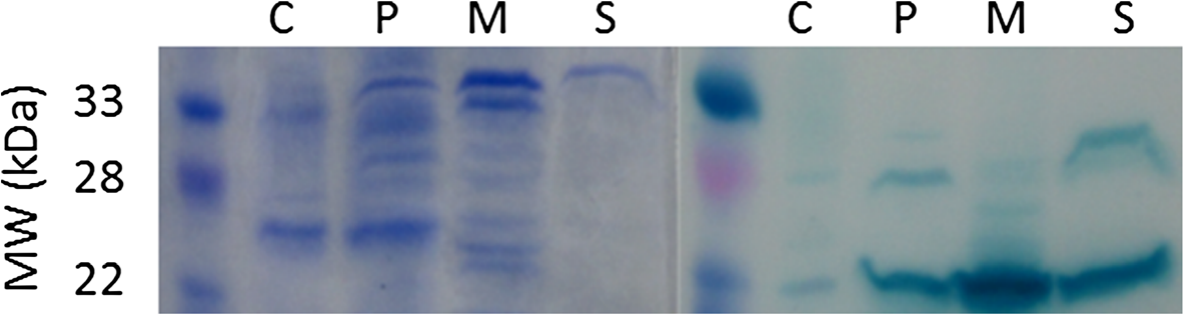
**SDS polyacrylamide gel and corresponding immunoblot of subcellular fractions for PhaP1:HlyA.** C – cytoplasmic fraction, P – periplasmic fraction, M – membrane fraction, S – concentrated supernatant (media) fraction. The position of phasin bands varies from roughly 22–26 kDa.

For *E. coli* cells expressing pCMEL1 and pLG575, a phasin band is observed at 22–26 kDa in the cytoplasmic fraction, the periplasmic fraction, the membrane fraction, and the concentrated extracellular media (Figure 1). This polyacrylamide gel and corresponding immunoblot demonstrated: 1) the ability for *E. coli* to produce non-codon optimized Phasin (from *R. eutropha)*, a protein not naturally expressed in *E. coli* and 2) translocation of phasin into different fractions of the cell. Compared to other studies that focused non-translocated phasin, the phasin band sizes in all of the different fractions are similar [19,29]. Since the translocation of phasin was successful, secretion of PHB using phasin was then attempted.

### Growth studies

**Figure 2:**
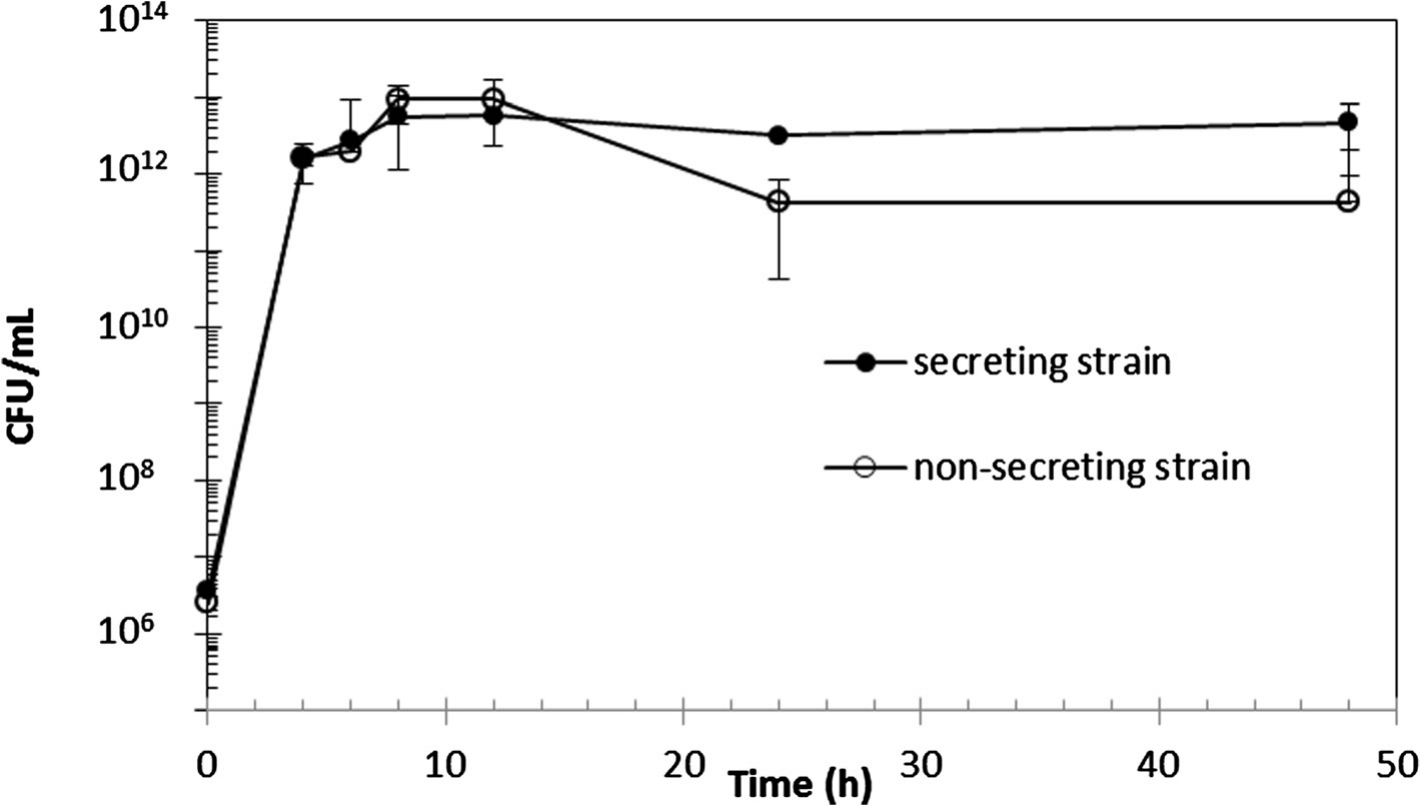
**CFU/mL vs. Time (hours) for secreting and non-secreting strains of PHB producing *E. coli*, averaged from triplicate experiments (one standard deviation shown).**

The PHB secreting strain consisted of pCMEL3 + pLG575, whereas the non-secreting strain consisted of pBHR68 + pLG575. From the CFU/mL vs. time graph (Figure 2) stationary phase was reached at approximately 8–12 h for both non-secreting and secreting strains. The non-secreting and secreting strains had the highest overall CFU/mL at approximately 9×10^12^ and 5×10^12^ CFU/mL, respectively, after 12 h. There was no significant difference between CFU/mL for the non-secreting and secreting strains after 24 and 48 h (p > 0.05). This statistical analysis on the CFU/mL demonstrated that there were no significant differences in *E. coli* growth between the two samples at times when PHB analysis was conducted (24 and 48 h). Furthermore, this suggests that the secretion of PHB does not affect cell viability.

### PHB production analysis

PHB production analysis was carried out 24 and 48 because after 24 h the *E. coli* harboring the plasmid systems were in stationary phase. Previous studies demonstrated that PHB did not accumulate to significant levels during the exponential growth phase. Acetyl-CoA is required for cell synthesis during the exponential phase but is diverted to produce PHB in the stationary phase, thus, there is a delay between carbon source utilization and PHB production [37].

PHB measured inside the cell is defined as the internal fraction and PHB collected by the CaCl_2_ precipitation method is the secreted fraction. 24 h after induction, the internal fraction of the cells demonstrated PHB production for both the secreting and non-secreting strains. While the non-secreting strain accumulated approximately 41.93 ± 13.5% of PHB in the dry cell weight at 24 h, the secreting strain accumulated approximately 28.85 ± 0.41% (Table 1). 48 h after induction the non-secreting and secreting strains had accumulated 47.24 ± 6.0% and 38.80 ± 15.5% PHB, respectively. There was no significant statistical difference seen in internally accumulated PHB in either the non-secreting or secreting strains at 24 and 48 h (p > 0.05) after induction. The internal levels of PHB accumulation in the non-secreting and secreting strains are comparable to those seen in other studies such as [38], where the authors showed accumulation of 42% PHB in *E. coli* DH5α harboring pBHR68 after 24–48 h [38].

**Table 1:** **Production of PHB in secreting (pCMEL3 + pLG575) and non-secreting (pBHR68 + pLG575) strains of *E. coli at *** **24 and 48 h**

		**% mass of PHB in dry mass**		**Production g/L PHB**			
**Strain**	**Time (h)**	**Secreted fraction**	**Non- secreted fraction**	**Secreted fraction**	**Non-secreted fraction**	**Total production**	**g PHB/g Glucose**
Non-secreting	24	0.31 ± 0.35	41.93 ± 13.5	0.40 ± 0.27	5.65 ± 1.1	6.05 ± 1.1	0.40 ± 0.07
	48	0.72 ± 0.89	47.24 ± 6.0	0.50 ± 0.41	5.43 ± 1.7	5.93 ± 1.8	0.40 ± 0.12
Secreting	24	0.69 ± 0.18	28.85 ± 0.41	0.40 ± 0.06	3.42 ± 0.33	3.82 ± 0.3	0.25 ± 0.02
	48	28.29 ± 7.2*	38.80 ± 15.5	2.57 ± 0.75*	4.58 ± 2.47	7.15 ± 2.6	0.48 ± 0.17

*Indicates statistical significance within column (p < 0.05).

PHB secreted fractions were analyzed at 24 and 48 h after induction for the secreting and non-secreting strains. PHB harvested in the secreted fraction of the non-secreting strains was 0.31 ± 0.35% and 0.72 ± 0.89%, respectively. The PHB present in the secreted phase demonstrated that some lysed cells containing PHB were found in this fraction after CaCl_2_ precipitation. This is to be expected from a differential centrifugation technique, such as that used in this study. 24 and 48 h after induction, the secreting strain produced 0.69 ± 0.18% and 28.29 ± 7.2% respectively in the secreted fraction. The level of PHB seen in the secreted fraction of the secreting strain was statistically significant after 48 h post induction, compared to the non-secreting strain (p < 0.05). This increase in PHB present in the secreted phase after 48 h indicates that the secretion system is functioning and producing higher amounts of PHB, with a small amount of non-secreted PHB ending up in the secreted fraction. These results demonstrate that PHB secretion is initiated 24 h after induction.

Secretion of PHB can help in downstream processes by aiding in PHB separation from biomass. Of the total PHB produced by the secreting strain after 48 h, 36% was collected in the secreted fraction and the remaining 64% was in the internal fraction. The secreting strain had a total PHB production of 7.15 g/L compared to 5.93 g/L for the non-secreting strain after 48 h post induction.

It has been demonstrated from previous studies that PHB can accumulate in larger quantities in *E. coli* when using a bioreactor compared to a shaker flask. A fed-batch bioreactor study by Choi et al. 1999, reported accumulation of up to 77% PHB of dry cell weight [39] and another study reported accumulation of up to 80% [37]. Future studies will be performed to determine how well the secreting strain performs under similar bioreactor growth conditions.

### SEM analysis

**Figure 3:**
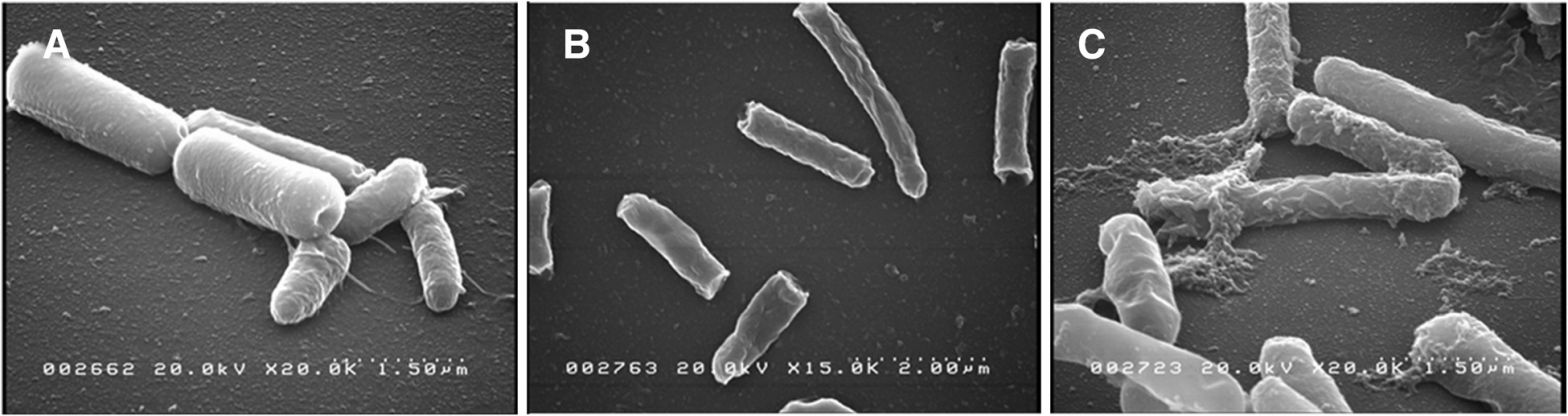
**SEM images taken from overnight cultures of *E. coli *** **XL1Blue harboring different plasmid systems: A) pBHR68 (non-secreting), B) pCMEL3 (non-secreting with phasin overexpression), and C) pCMEL3 + pLG575 (complete PHB production and secretion system).**

SEM is not widely used for PHB analysis since a surface topographical analysis is typically of little use. SEM has however been used to visualize PHA granules produced from recombinant *E. coli*[40] and PHA degradation from a variety of organisms [41]. In the case of secretion, SEM can provide images of what is occurring at the surface of *E. coli* during the secretion process. Figure 3A shows a PHB non-secreting *E. coli* strain harboring the pBHR68 plasmid. Figure 3B shows *E. coli* that is accumulating PHB and overexpressing phasin. Figure 3C shows the full secretion system in *E. coli* (pCMEL3 + pLG757). Figure 3C suggests that PHB is being secreted outside of the bacteria and into the medium. These observations further demonstrate the functioning of the PHB secretion system.

The SEM photo in Figure 3C suggests that secretion of PHB occurs at the polar regions of the cell. Interestingly it has been found in previous studies [42,43] that PHB granule formation occurs at the cell poles in *E. coli* when both phasin and PHB are being produced. When PHB is being produced the interpretation is that PHA synthase is active, and it has previously been suggested that PHA synthase has polar targeting information [43].

It is interesting that *E. coli* is able to secrete PHB through pore sizes of 3.5 nm [36] in diameter. From the results of this this study not all the PHB produced is being secreted, suggesting there could be differences between the secreted and non-secreted PHB.

## Conclusions

This study demonstrated the successful expression of phasin, PhaP1*,* and its translocation using type I secretion in *E. coli*. Once translocation of PhaP1 was successful, a recombinant synthetic biological system to secrete PHBs was designed and tested. Initiation of PHB secretion occurs between 24 and 48 h after induction of PHB synthesis. Of the total PHB produced by the secreting strain, 36% was collected in the secreted fraction and 64% remained in the internal fraction after 48 h.

Secretion of PHB should help in downstream processing whereby PHB is separated from the cell mass, which can aid in PHB recovery and purification. Future studies will include a detailed comparison of secreted versus non-secreted PHB characteristics.

## Methods

### Strains and plasmids

Genetic parts were constructed in accordance with the BioBrick and BioFusion technical standards [44,45]. The BioFusion standard was specifically used to create phasin and signal peptide parts because this genetic fusion system is compatible with the original BioBrick standard [45,46].

Descriptions of strains and plasmids used to study PHB and phasin production and translocation are provided as Table 2. Completed BioBrick parts were transformed in BL21-Gold (DE3) competent *E. coli* (Agilent Technologies, Santa Clara, CA) for protein expression studies. PHB production and secretion studies were carried out in *E. coli* XL1 Blue (Agilent Technologies, Santa Clara, CA). Plasmid pLG575 includes the coding regions for proteins HlyB and HlyD [47,48]. Plasmids pSB1AK3, pSB1A3, and pSB3K3 are BioBrick standard vectors for assembly and expression of BioBrick genetic devices [49].

**Table 2:** Strains, plasmids, and oligonucleotides used in this study

**Strains**	**Relevant characteristics**	**Reference**
**Strains**		
BL21-Gold (DE3)	E. coli B F^–^ ompT hsdS(rB^–^ mB^–^) dcm^+^ Tet^R^ gal λ(DE3) endA Hte	Agilent technologies
XL1 Blue	recA1 endA1 gyrA96 thi-1 hsdR17 supE44 relA1 lac [F’ proAB lacIqZΔM15 Tn10 (Tetr)]	Agilent technologies
Cupriavidus necator H16	Wild type, PHA producing	ATCC 17699
**Plasmids**		
pLG575	pACYC184 derivative, HlyBD, p15A origin, Cm^R^	[47]
pBHR68	pBluescript SK-, phbCAB genes from R. eutropha	[16]
pSB1AK3	High copy BioBrick vector, pMB1 origin, Amp^R^ and Kan^R^	[49]
pSB1A3	High copy BioBrick vector, pMB1 origin, Amp^R^	[49]
pSB3K3	Medium copy BioBrick standard vector, p15A origin, Kan^R^	[49]
pCMEL1	phaP1, C-terminal BioFusion with HlyA signal peptide, Lac promoter (BBa_R0010), RBS(BBa_B0034), in pSB1A3	This study
pCMEL2	phaP1, C-terminal BioFusion with HlyA signal peptide, Lac promoter (BBa_R0010), RBS(BBa_B0034), in pSB3K3	This study
pCMEL3	phaP1, C-terminal BioFusion with HlyA signal peptide, Lac promoter (BBa_R0010), RBS(BBa_B0034), in pBHR68	This study
**Oligonucleotides**		
PhaP1FOR	**5′-**gaattcgcggccgcttctagaatgatcctcaccccggaaca**-3**′	This study
PhaP1REV	**5′-** ctgcagcggccgctactagttcaggcagccgtcgtcttct-**3′**	This study
g114t	**5′-**cgtcgagctgaaccttcaggtcgtcaagact**-3′**	This study
g114t_antisense	**5′**-agtcttgacgacctgaaggttcagctcgacg-**3′**	This study

### BioBrick and plasmid construction

All restriction enzymes and related reagents were purchased from Thermo Fisher Scientific Inc. (Glen Burnie, MD). The signal peptide HlyA was made through synthetic design and construction as mention in Linton et al. 2012 (DNA 2.0, Menlo Park, CA) [35]. A BioFusion-compatible phasin BioBrick was constructed by isolating phaP1 from the genomic DNA of *R. eutropha* using PCR with primers PhaP1FOR and PhaP1REV that included the BioFusion prefix and suffix as overhanging ends (Table 2). The 620 bp PCR product was isolated by gel electrophoresis, digested with EcoRI and SpeI, and ligated into pSB3K3. A PstI site was removed from phaP1 (while conserving amino acid sequence) using a QuikChange II Site-Directed Mutagenesis Kit and the QuikChange® Primer Design Program (Agilent Technologies, Santa Clara, CA). The designed primers (g114t and g114t_antisense) for site-directed mutagenesis are shown in Table 2. The PstI mutation in phaP1 was successfully carried out and confirmed by sequence analysis.

**Figure 4:**
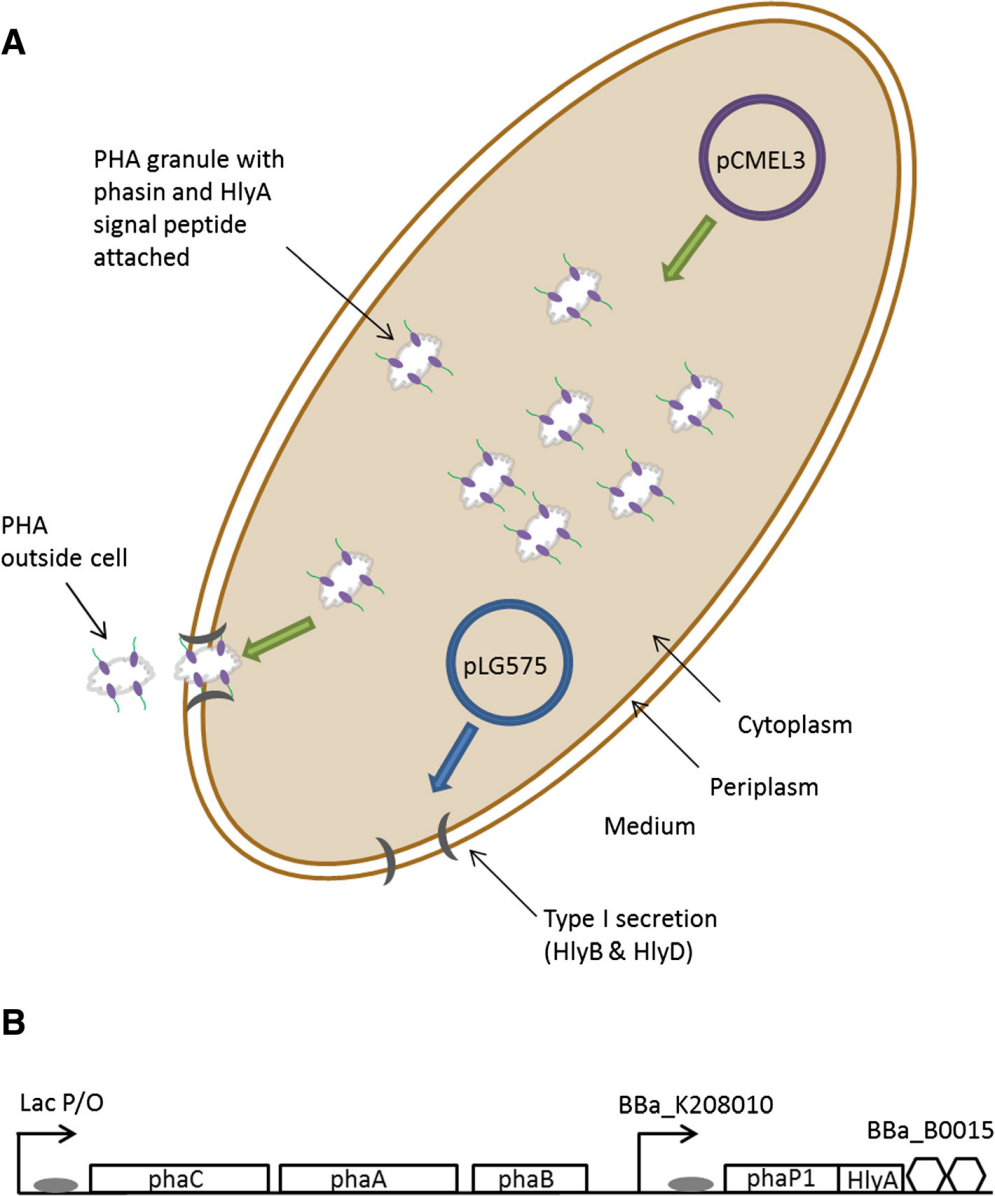
**PHB secretion. A**. Schematic for PHB secretion containing dual plasmid system pCMEL3 and pLG575. Phasin with attached signal peptide binds to PHB granule surface and the PHB-phasin-signal peptide complex is targeted for type I secretion. **B**. pCMEL3 plasmid consisting of phaC, phaC, and phaB genes from pBHR68. pCMEL3 also contains the genes needed for phasin-HylA production.

Step-wise assembly of composite BioBrick devices was primarily carried out in pSB1AK3. Completed devices were subsequently ligated into pSB1A3 and pSB3K3. The lac promoter (BBa_R0010) and ribosome binding site (BBa_B0034) were used as described in Linton et al. 2012 [35]. The pCMEL1 plasmid was used for studies on phasin translocation because its origin of replication (pMB1) was compatible with the origin of replication of pLG575 (p15A). BL21-Gold (DE3) was co-transformed with pCMEL1 and pLG575. The pCMEL3 plasmid was used for studies on type I secretion of PHA. The composite part containing the promoter, RBS, coding region, and terminator were cloned into pBHR68 from pCMEL2 by digestion with EcoRI and XhoI. XL1-Blue was co-transformed with pCMEL3 and pLG575 for PHA secretion studies. A schematic of the secretion system is presented in Figure 4.

### Cellular fractionation and western blotting

Overnight cultures of *E. coli* BL21-Gold (DE3) harboring pCMEL1 and pLG575 were used to inoculate (1:100 v/v dilution) 100 ml of LB media containing 25 μg/ml chloramphenicol (Acros Organics, Fair Lawn, NJ) and 50 μg/ml ampicillin (IBI Scientific, Peosta, IA) [50]. The lysozyme/EDTA/osmotic shock and chloroform-based cellular fractionation procedures and methods for analyzing supernatant fractions are previously discussed in Linton et al. 2012 [35]. Briefly, 50 mL of cell culture were centrifuged and the pellet was resuspended in a buffer and subjected to osmotic shock. Centrifugation was used to separate the periplasm from spheroplast. Cytoplasm and membranes were fractionated via ultracentrifugation.

Approximately 40 μg of protein from each of the respective fractioned samples were separated using precast 12% SDS-polyacrylamide tris-glycine gels (Jule Inc., Milford, CT). Electrophoresis operating conditions were used as specified by the manufacturer. Phasin immunoblotting was carried out using a PhaP1 specific antibody [51,52]. An Anti-Rabbit IgG HRP conjugated secondary antibody was purchased from Promega (Madison, WI). Respective primary and secondary antibody concentrations of 1:50,000 v/v and 1:2,500 v/v were used.

### PHB secretion studies

All secretion studies were carried out in triplicate. Secretion studies were conducted with the secreting system (pCMEL3 + pLG575 in XL1 Blue) and non-secreting system (pBHR68 + pLG575 in XL1 Blue). The non-secreting system does not contain phasin-HlyA.

### Media formulation and growth conditions

*R. eutropha* was cultured following the methods outlined in Linton et al. 2012 [53]. M9 salts (Becton, Dickinson and Co, Sparks, MD) supplemented with 1.5% (w/v) glucose (ACS grade, Acros Organics, Fair Lawn, NJ) and 0.2% (w/v) yeast extract (Becton, Dickinson and Co, Sparks, MD) was used for PHB secretion studies in *E. coli*[38]. Overnight *E. coli* cultures harboring specific plasmids were inoculated from freezer stocks into 5 ml of M9 media with chloramphenicol (34 μg/ml), ampicillin (50 μg/ml), and grown in an orbital shaker table at 220 rpm at 37°C. Overnight cultures were then used to seed larger 250 ml flasks (50 ml media volume) at an initial optical density (OD_600_) of 0.05 at time 0 h. 0.1 mM Isopropyl β-D-1-thiogalactopyranoside (IPTG) (Gold Biotechnology, Inc. St. Louis, MO) was added to each flask at time 0 h. Flasks were removed at 24, and 48 h and analyzed for PHB. CFU/mL was measured at time points 0, 4, 6, 8, 12, and 24 h.

### Recovery of secreted PHB

PHB granules can agglomerate together naturally and previous studies have demonstrated the use of Calcium chloride (CaCl_2_) to enhance this process. A study by Fidler et al. used CaCl_2_ to purify PHB from lysed cells by selectively aggregating PHB granules. It was observed that PHB granules fell to the bottom of the test tube after addition of CaCl_2_[54]. Another CaCl_2_ method for PHB recovery by Resch et al. used a low speed centrifugation step to further enhance PHB recovery from cell debris [55].

In this study, techniques for secreted PHB recovery were adapted from the methods outlined in [55]. At 24, and 48 h 0.01 M CaCl_2_ (final concentration, Avantor Performance Materials, Inc. Center Valley, PA) was added to the bacterial culture and mixed by inverting the tube several times. The tubes were then allowed to sit for 10 mins at room temperature and then centrifuged at 54 × g for 5 min. The supernatant was removed and transferred to a fresh tube and the pellet was freeze dried. The supernatant was centrifuged at 3452 × g for 10 mins and the pellet was freeze dried. The pellet from the first centrifugation contained secreted PHB with CaCl_2_ and the pellet from the second centrifugation contained bacterial mass and non-secreted PHB. Secretion studies and PHB analysis were conducted in triplicate.

### PHB concentration determination

PHB concentrations were determined based on a NMR-GC method described in Linton et al. 2012 [53]. Briefly, samples were lyophilized after which approximately 15 mg of sample was mixed with equal volumes of sodium hypochlorite and deuterated chloroform. Samples were centrifuged and 1H NMR was carried out on the PHB fraction. PHB concentration was determined from a NMR-GC standard.

### Scanning electron microscopy (SEM)

To visually show PHB secretion from *E. coli*, SEM was performed. SEM protocols were used as mentioned in [56]. Briefly, secreting and non-secreting strains were grown up overnight and fixed onto glass cover slips. Samples were mounted on aluminum stubs and sputter coated with 10 nm gold. SEM was carried out using a Hitachi S4000 SEM.

### Statistical analysis

All growth (CFU/mL) and PHB yield studies were carried out in triplicate to show consistency of data. Statistical analysis was conducted with Statistical Analysis Software (SAS 9.3, SAS Institute Inc., Cary, NC). A two-way analysis of variance (ANOVA) with tukey post hoc comparison performed on significant results (confidence level 95%).

## Competing interests

The authors declare that they have no competing interests.

## Authors’ contributions

AR and EL conducted experiments and wrote the manuscript draft. CDM conceived and designed the experiments and wrote the manuscript. AH assisted with experiments and data analysis. RCS assisted with experimental design and manuscript revision. All authors read and approved the final manuscript.
